# Supplementary material for: Group-Based Trajectory Modeling of N-Terminal Pro-Brain Natriuretic Peptide Levels in Pulmonary Artery Hypertension Associated with Connective Tissue Disease
Source: Healthcare (Basel). 2024 Aug 16;12(16):1633. doi: 10.3390/healthcare12161633 (PMC11354151; doi:10.3390/healthcare12161633)
Supplement: Supplementary file 1 [file healthcare-12-01633-s001.zip › Figure S1.pdf]

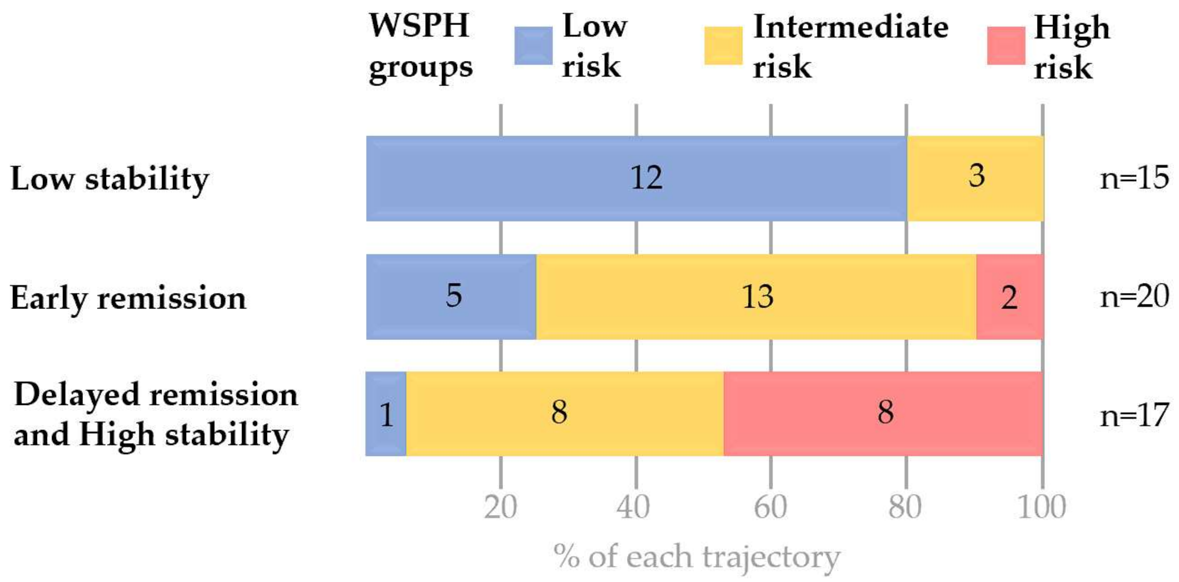

Figure S1. Distribution of WSPH groups across NT-proBNP trajectories. Abbreviations: WSPH, World Symposium on Pulmonary Hypertension.
