# Supplementary material for: Group-Based Trajectory Modeling of N-Terminal Pro-Brain Natriuretic Peptide Levels in Pulmonary Artery Hypertension Associated with Connective Tissue Disease
Source: Healthcare (Basel). 2024 Aug 16;12(16):1633. doi: 10.3390/healthcare12161633 (PMC11354151; doi:10.3390/healthcare12161633)
Supplement: Supplementary file 1 [file healthcare-12-01633-s001.zip › Table S2.pdf]

Table S2. Baseline clinical characteristics of NT-proBNP trajectories.

Baseline clinical characteristics	All (n=52)	NT-proBNP trajectories			
		Low stability (n=15)	Early remission (n=20)	Delayed remission (n=11)	High stability (n=6)
Female, n (%)	52 (100.00)	15 (100.00)	20 (100.00)	11 (100.00)	6 (100.00)
Age, years	41.00 (34.00, 55.25)	46.00 (35.00, 60.00)	34.00 (25.00, 45.00)	49.00 (37.00, 60.00)	47.00 (35.75, 56.75)
Primary CTD					
SLE, n (%)	22 (42.30)	8 (53.32)	8 (40.00)	6 (54.55)	0 (0.00)
pSS, n (%)	13 (25.00)	4 (26.67)	5 (25.00)	3 (27.27)	1 (16.67)
SSc, n (%)	6 (11.54)	1 (6.67)	0 (0.00)	2 (18.18)	3 (50.00)
RA, n (%)	2 (3.85)	1 (6.67)	1 (5.00)	0 (0.00)	0 (0.00)
MCTD, n (%)	3 (5.77)	0 (0.00)	2 (10.00)	0 (0.00)	1 (16.67)
UCTD, n (%)	6 (11.54)	1 (6.67)	4 (20.00)	0 (0.00)	1 (16.66)
CTD duration, years	0.00 (0.00, 6.50)	4.00 (0.00, 7.00)	0.00 (0.00, 0.00)	1.00 (0.00, 8.00)	2.00 (0.75, 8.75)
Active CTD, n (%)	23 (44.23)	6 (40.00)	11 (55.00)	4 (36.36)	2 (33.33)
Intensive CTD immunotherapy, n (%)	39 (75.00)	11 (73.33)	19 (95.00)	7 (63.64)	2 (33.33)
PAH duration, years	1.00 (0.00, 2.00)	1.00 (0.00, 2.00)	0.00 (0.00, 1.00)	1.00 (0.00, 2.00)	1.00 (0.00, 2.25)
WSPH groups					
Low risk, n (%)	18 (34.62)	12 (80.00)	5 (25.00)	0 (0.00)	1 (16.67)
Intermediate risk, n (%)	24 (46.15)	3 (20.00)	13 (65.00)	6 (54.55)	2 (33.33)
High risk, n (%)	10 (19.23)	0 (0.00)	2 (10.00)	5 (45.45)	3 (50.00)
6MWD, m	457.00 (359.75, 524.25)	500.00 (457.00, 570.00)	458.50 (392.75, 538.75)	380.00 (227.00, 428.00)	266.00 (202.50, 473.25)
WHO-FC					
I, n (%)	2 (3.85)	1 (6.67)	1 (5.00)	0 (0.00)	0 (0.00)
II, n (%)	24 (46.15)	11 (73.33)	10 (50.00)	2 (18.18)	1 (16.67)
III, n (%)	23 (44.23)	3 (20.00)	9 (45.00)	7 (63.64)	4 (66.67)
IV, n (%)	3 (5.77)	0 (0.00)	0 (0.00)	2 (18.18)	1 (16.67)
RAD, mm	40.00 (37.00, 45.75)	36.00 (34.00, 38.00)	40.00 (38.25, 44.75)	47.00 (42.00, 51.00)	45.50 (41.00, 51.25)
RVDd, mm	42.12±5.59	38.00±3.91	41.80±4.07	45.73±6.54	46.83±4.49
TRV, cm/s	398.90±65.05	367.33±44.48	401.00±72.25	427.55±65.37	418.33±63.06
TAPSE/PASP ratio	0.25±0.12	0.29±0.14	0.27±0.12	0.19±0.10	0.20±0.09
Pericardial effusion, n (%)	25 (48.08)	5 (33.33)	7 (35.00)	8 (72.73)	5 (83.33)
mPAP, mmHg	41.00 (34.25, 55.75)	36.00 (26.00, 40.00)	41.00 (34.25, 57.75)	49.00 (42.00, 70.00)	53.50 (32.25, 58.50)
SVO_2, %	65.00 (57.25, 71.00)	70.00 (67.00, 75.00)	63.50 (59.25, 67.75)	52.00 (44.00, 71.00)	64.00 (54.50, 71.75)
mRAP, mmHg	5.00 (3.00, 8.75)	3.00 (2.00, 5.00)	5.50 (3.00, 9.00)	7.00 (5.00, 9.00)	5.50 (3.00, 9.75)
PAWP, mmHg	8.27±2.97	7.40±2.69	8.20±3.02	10.36±2.84	6.83±2.14
mRVP, mmHg	24.00 (19.00, 33.50)	19.00 (16.00, 25.00)	22.50 (19.50, 34.75)	26.00 (25.00, 40.00)	29.50 (16.75, 36.00)
PVR, Wood	7.42 (4.63, 12.43)	4.43 (3.27, 5.93)	8.20 (5.55, 13.13)	10.63 (7.44, 20.00)	13.18 (5.31, 28.71)
Cardiac index, L/min/m^2	2.92±0.88	3.64±0.61	2.72±0.75	2.65±0.76	2.25±1.13
PAH targeted drug therapy					
ERA, n (%)	3 (5.77)	3 (20.00)	0 (0.00)	0 (0.00)	0 (0.00)
PDE5i, n (%)	3 (5.77)	2 (13.33)	0 (0.00)	1 (9.10)	0 (0.00)
ERA+PDE5i, n (%)	30 (57.69)	8 (53.33)	12 (60.00)	6 (54.55)	4 (66.67)
ERA+PRA, n (%)	1 (1.92)	0 (0.00)	0 (5.00)	1 (9.10)	0 (0.00)
PDE5i+PRA, n (%)	1 (1.92)	1 (6.67)	0 (0.00)	0 (0.00)	0 (0.00)
ERA+PDE5i+PRA, n (%)	14 (26.92)	1 (6.67)	8 (35.00)	3 (27.27)	2 (33.33)

Abbreviations: NT-proBNP, N-terminal pro-brain natriuretic peptide; SLE, systemic lupus erythematosus; pSS, primary Sjogren's syndrome; SSc, systemic sclerosis; RA, rheumatoid arthritis; MCTD, mixed connective tissue disease; UCTD, undifferentiated connective tissue disease; CTD, connective tissue disease; PAH, pulmonary artery hypertension; 6MWD, 6-minite walk distance; WHO-FC, World Health Organization functional class; RAD, right atrial diameter; RVDd, right ventricular end diastolic diameter; TRV, tricuspid regurgitation velocity; TAPSE, tricuspid annular plane systolic excursion; PASP, pulmonary artery systolic pressure; mPAP, mean pulmonary artery pressure; SVO_2, oxygen saturation of mixed venous blood; mRAP, mean right atrial pressure; PAWP, pulmonary artery wedge pressure; mRVP, mean right ventricular pressure; PVR, pulmonary vascular resistance; ERA, endothelin receptor antagonists; PDE5i, phosphodiesterase 5 inhibitors; PRA, prostacyclin receptor agonist.
