# Supplementary material for: Group-Based Trajectory Modeling of N-Terminal Pro-Brain Natriuretic Peptide Levels in Pulmonary Artery Hypertension Associated with Connective Tissue Disease
Source: Healthcare (Basel). 2024 Aug 16;12(16):1633. doi: 10.3390/healthcare12161633 (PMC11354151; doi:10.3390/healthcare12161633)
Supplement: Supplementary file 1 [file healthcare-12-01633-s001.zip › Table S1.pdf]

Table S1. Eligible GBTM models.

Model	*p*	APP	Proportions, %	BIC	OCC	E_j
3_310	0.039-0.000-0.000	0.93-0.85-0.93	67.31-23.08-9.62	-207.95/-201.36	6.05-18.70-122.52	0.816
3_210	0.002-0.000-0.000	0.96-0.81-0.93	59.62-30.77-9.62	-207.47/-201.54	14.49-9.46-134.88	0.797
3_200	0.000-0.000-0.000	0.98-0.93-1.00	38.46-51.92-9.62	-188.44/-183.17	99.41-11.84-10907.52	0.893
3_120	0.008-0.000-0.000	0.96-1.00-1.00	53.85-36.54-9.62	-187.63/-181.71	20.90-412.67-16150.64	0.932
3_100	0.000-0.000-0.000	0.95-0.89-0.97	46.15-44.23-9.62	-207.14/-202.53	20.31-10.19-298.89	0.820
3_020	0.000-0.000-0.000	0.93-0.98-1.00	51.92-38.46-9.62	-188.44/-183.17	11.84-99.40-10907.52	0.893
3_010	0.000-0.000-0.000	0.89-0.95-0.97	44.23-46.15-9.62	-207.14/-202.53	10.19-20.31-298.89	0.820
4_3200	0.000-0.000-0.000-0.000	0.88-0.92-1.00-0.97	38.46-21.15-28.85-11.54	-190.67/-182.11	11.53-45.64-1104.02-228.74	0.865
4_3101	0.014-0.036-0.000-0.000	0.95-0.93-0.98-0.85	25.00-46.15-9.62-19.23	-190.53/-181.97	60.67-14.44-535.66-23.31	0.852
4_3100	0.001-0.000-0.000-0.000	0.89-0.87-1.00-0.98	40.38-21.15-28.85-9.62	-188.78/-180.88	12.12-25.14-6094.15-423.30	0.859
4_3010	0.001-0.000-0.000-0.000	0.89-1.00-0.87-0.98	40.38-28.85-21.15-9.62	-188.78/-180.88	12.12-6094.15-25.14-423.30	0.859
4_3002	0.000-0.000-0.000-0.000	0.88-1.00-0.97-0.92	38.46-28.85-11.54-21.15	-190.67/-182.11	11.53-1104.02-228.74-45.64	0.865
4_3001	0.001-0.000-0.000-0.000	0.89-1.00-0.98-0.87	40.38-28.85-9.62-21.15	-188.78/-180.88	12.12-6094.15-423.29-25.14	0.859
4_2102	0.003-0.007-0.000-0.000	0.96-0.96-1.00-1.00	3.85-50.00-9.62-36.54	-191.73/-183.17	644.28-22.97-22899.64-1167.86	0.952
4_2101	0.000-0.014-0.000-0.000	0.90-0.97-0.98-0.75	28.85-46.15-9.62-15.38	-191.05/-183.15	23.41-44.30-598.32-16.66	0.863
4_2100	0.000-0.000-0.000-0.000	0.93-0.80-0.92-0.98	26.92-17.31-46.15-9.62	-191.42/-184.18	34.69-19.54-12.76-555.63	0.832
4_2001	0.000-0.000-0.000-0.003	0.99-0.90-1.00-0.77	36.54-28.85-9.62-25.00	-191.89/-184.65	186.97-21.38-35598.15-9.96	0.818
4_2000	0.000-0.000-0.000-0.000	0.96-0.92-0.84-0.99	40.38-46.15-3.85-9.62	-191.92/-185.34	36.52-14.16-134.14-1802.89	0.887
4_1310	0.036-0.014-0.000-0.000	0.93-0.95-0.85-0.98	46.15-25.00-19.23-9.62	-190.53/-181.97	14.44-60.67-23.31-535.66	0.852
4_1301	0.036-0.014-0.000-0.000	0.93-0.95-0.98-0.85	46.15-25.00-9.62-19.23	-190.53/-181.97	14.44-60.67-535.66-23.31	0.852
4_1300	0.000-0.001-0.000-0.000	0.87-0.89-1.00-0.98	21.15-40.38-28.85-9.62	-188.78/-180.88	25.14-12.12-6094.15-423.29	0.859
4_1220	0.007-0.003-0.000-0.000	0.96-0.96-1.00-1.00	50.00-3.85-36.54-9.62	-191.73/-183.17	22.97-644.28-1167.82-22899.64	0.952
4_1210	0.014-0.000-0.000-0.000	0.97-0.90-0.75-0.98	46.15-28.85-15.38-9.62	-191.05/-183.15	44.30-23.41-16.66-598.32	0.863

Table S1. *Cont.*

Model	*p*	APP	Proportions, %	BIC	OCC	E_j
4_1201	0.014-0.000-0.000-0.000	0.97-0.90-0.98-0.75	46.15-28.85-9.62-15.38	-191.05/-183.15	44.30-23.41-598.32-16.66	0.863
4_1200	0.003-0.000-0.000-0.000	0.77-0.99-0.90-1.00	25.00-36.54-28.85-9.62	-191.89/-184.65	9.96-186.97-21.38-35598.15	0.818
4_1130	0.000-0.036-0.014-0.000	0.85-0.93-0.95-0.98	19.23-46.15-25.00-9.62	-190.53/-181.97	23.31-14.44-60.67-535.68	0.852
4_1103	0.036-0.000-0.000-0.014	0.93-0.85-0.98-0.95	46.15-19.23-9.62-25.00	-190.53/-181.97	14.44-23.31-535.67-60.67	0.852
4_1100	0.046-0.000-0.000-0.000	0.95-0.88-0.66-0.94	46.15-40.38-3.85-9.62	-212.88/-206.30	20.87-10.96-48.12-137.19	0.813
4_1020	0.003-0.000-0.000-0.000	0.77-0.90-0.99-1.00	25.00-28.85-36.54-9.62	-191.89/-184.65	9.96-21.38-186.97-35598.15	0.818
4_1002	0.003-0.000-0.000-0.000	0.77-0.90-1.00-0.99	25.00-28.85-9.62-36.54	-191.89/-184.65	9.96-21.38-35598.15-186.97	0.818
4_0320	0.000-0.000-0.000-0.000	1.00-0.88-0.92-0.97	28.85-38.46-21.15-11.54	-190.67/-182.11	1104.02-11.53-45.64-228.74	0.865
4_0310	0.000-0.001-0.000-0.000	1.00-0.89-0.87-0.98	28.85-40.38-21.15-9.62	-188.78/-180.88	6094.14-12.12-25.14-423.29	0.859
4_0302	0.000-0.000-0.000-0.000	1.00-0.88-0.97-0.92	28.85-38.46-11.54-21.15	-190.67/-182.11	1104.02-11.53-228.74-45.64	0.865
4_0301	0.000-0.001-0.000-0.000	1.00-0.89-0.98-0.87	28.85-40.38-9.62-21.15	-188.78/-180.88	6094.14-12.12-423.29-25.14	0.859
4_0210	0.000-0.000-0.000-0.000	0.92-0.93-0.80-0.98	46.15-26.92-17.31-9.62	-191.42/-184.18	12.76-34.69-19.54-555.64	0.832
4_0201	0.000-0.000-0.000-0.000	0.92-0.93-0.98-0.80	46.15-26.92-9.62-17.31	-191.42/-184.18	12.76-34.69-555.64-19.54	0.832
4_0200	0.000-0.000-0.000-0.000	0.84-0.96-0.92-0.99	3.85-40.38-46.15-9.62	-191.92/-185.34	134.14-36.53-14.16-1802.85	0.887
4_0120	0.000-0.003-0.000-0.000	0.90-0.77-0.99-1.00	28.85-25.00-36.54-9.62	-191.89/-184.65	21.38-9.96-186.97-35598.15	0.818
4_0102	0.000-0.003-0.000-0.000	0.90-0.77-1.00-0.99	28.85-25.00-9.62-36.54	-191.89/-184.65	21.38-9.96-35598.15-186.97	0.818
4_0020	0.000-0.000-0.000-0.000	0.84-0.92-0.96-0.99	3.85-46.15-40.38-9.62	-191.92/-185.34	134.14-14.16-36.53-1802.87	0.887
4_0002	0.000-0.000-0.000-0.000	0.92-0.84-0.99-0.96	46.15-3.85-9.62-40.38	-191.92/-185.34	14.16-134.14-1802.85-36.53	0.887

Abbreviations: APP, average posterior probability; BIC, Bayesian information criterion; OCC, odds of correct classification; Ej, relative entropy.
